# *Plasmodium falciparum* with Multidrug Resistance 1 Gene Duplications, Senegal

**DOI:** 10.3201/eid1905.121603

**Published:** 2013-05

**Authors:** Aurélie Pascual, Bécaye Fall, Nathalie Wurtz, Mansour Fall, Cheikhou Camara, Aminata Nakoulima, Eric Baret, Bakary Diatta, Boubacar Wade, Sébastien Briolant, Bruno Pradines

**Affiliations:** Institut de Recherche Biomédicale des Armées, Marseille, France (A. Pascual, N. Wurtz, E. Baret, S. Briolant, B. Pradines);; Aix Marseille Université, Marseille (A. Pascual, N. Wurtz, E. Baret, S. Briolant, B. Pradines);; Centre National de Référence du Paludisme, Marseille (A. Pascual, B. Pradines);; Hôpital Principal de Dakar, Dakar, Sénégal (B. Fall, M. Fall, C. Camara, A. Nakoulima, B Diatta, B. Wade, B. Pradines)

**Keywords:** Plasmodium falciparum, Plasmodium falciparum multidrug resistance 1, genes, Pfmdr1, malaria, parasites, antimicrobial resistance, Senegal, Africa

**To the Editor**: Amplification and overexpression of the *Plasmodium falciparum* multidrug resistance 1 gene (*Pfmdr1*) have been associated with mefloquine resistance in *P. falciparum* malaria in Asia (*1*). Amplification of *Pfmdr1* in Africa has occurred rarely. Only 12 isolates with >2 copies of *Pfmdr1* were identified in Africa during 1993–2012: 3 in Côte d’Ivoire (*2*,*3*), 1 in Burkina Faso (*3*), 1 in Togo (*3*), 3 in eastern Sudan (*4*), 2 in Kenya (*5*,*6*), and 1 Senegal (*7*). Another isolate was obtained in a patient from Benin who did not respond clinically to mefloquine treatment (*8*). *Pfmdr1* amplification has not been found in samples collected either before or after treatment for recurring *P. falciparum* infection in Africa in many studies.

In Dakar, Senegal, and its surrounding suburbs, malaria is transmitted with spatial heterogeneity to the human mosquito bite rate, which ranged from 0.1 to 250 bites per person per night during the rainy seasons of 2007–2010. *P. falciparum* isolates from patients with malaria who lived in Dakar (>80%) and the surrounding area and did not travel during the previous month were obtained during the rainy seasons of October 2009–January 2010 (172 patients, 42% female) and August 2010–January 2011 (129 patients, 38% female). Informed verbal consent from the patients and/or their parents/guardians was obtained before blood collection; the study was approved by the ethical committee of the Hôpital Principal de Dakar. 

Of the 301 patients, 54% were recruited from the emergency department during each of the 2 seasons; other patients were recruited from the intensive care unit (18% during October 2009–January 2010 and 20% during August 2010–January 2011), pediatric department (9% and 5%), and other units (19% and 21%). No significant differences were found between the 2 seasons for parasitemia (p = 0.160), sex ratio (p = 0.446), living area (p = 0.651), or hospital admission status (p = 0.567). Information on antimalarial treatment before admission was not available.

We analyzed the *Pfmdr1* copy number for the 301 isolates by using TaqMan real-time PCR as described (*7*); 167 isolates were successfully evaluated by using the 72-h histidine-rich protein 2 test as described (*9*). *P. falciparum* clones 3D7 (1 *Pfmdr* copy) and W2 (3 *Pfmdr1* copies) were used as controls for the determination of the *Pfmdr1* copy number and for the validation of the batches of plates used in the susceptibility tests.

A total of 9 isolates with 2 *Pfmdr1* copies were identified, 1 collected during the 2009–2010 season and 8 collected during the 2010–2011 season. This finding reflects a statistically significant 10-fold increase in frequency of isolates from 1 season to the next (p = 0.0057 by Fisher exact test). All of the isolates with duplicated copies had 1 allelic family for each of the 3 genes (*msp1*, *msp2*, and *glurp*), confirming that these infections were single and not mixed. 

We did not find an association between *Pfmdr1* copy number and the 50% inhibitory concentration values for mefloquine (p = 0.345), monodesethylamodiaquine (p = 0.729), lumefantrine (p = 0.314) and chloroquine (p = 0.579), but a significant association was found for dihydroartemisinin (p = 0.003). Odds ratio for in vitro reduced susceptibility to dihydroartemisinin associated with 2 *Pfmdr1* copies was 1.4. Although the number of isolates with 2 *Pfmdr1* copies we obtained is small, these data are consistent with previous reports on *Pfmdr1* copies and in vitro responses to artemisinin derivatives (*10*). 

The role of the amplification of *Pfmdr1* in *P. falciparum* resistance to antimalarial drugs in Africa is debated. In our study, we identified as many samples with multiple *Pfmdr1* copies (9) as had been identified during the previous 19 years. The 9 patients from whom these isolates were collected were successfully treated with quinine. One study found clinical treatment failure for mefloquine in Africa was associated with in vitro resistance and amplification of *Pfmdr1* (*8*). In addition, amodiaquine resistance is not related to the amplification of *Pfmdr1* (*6*). In Sudan, 3 isolates with 2 *Pfmdr1* copies were identified in patients before and after treatment with artemether-lumefantrine during the 28-day follow-up period, but these patients were classified as having adequate clinical and parasitologic responses (*4*). 

The increased prevalence of *Pfmdr1* duplication in *P. falciparum* isolates from patients in Dakar within a 2-year period is cause for concern and vigilance. The presence of these duplicated *Pfmdr1* copies could be associated with rare clinical failures of *P. falciparum* infections to respond to mefloquine treatment or artemisinin-based combination therapy. However, our findings highlight the need for active surveillance of the prevalence of *Pfmdr1* duplication in *P. falciparum* isolates and for ex vivo and in vivo studies in Senegal and in other parts of Africa.
